# Classification of patients with knee osteoarthritis in clinical phenotypes: Data from the osteoarthritis initiative

**DOI:** 10.1371/journal.pone.0191045

**Published:** 2018-01-12

**Authors:** A. Dell’Isola, M. Steultjens

**Affiliations:** Institute of Applied Health Research/ School of Health and Life Sciences, Glasgow Caledonian University, Glasgow, Scotland; University of Umeå, SWEDEN

## Abstract

**Objectives:**

The existence of phenotypes has been hypothesized to explain the large heterogeneity characterizing the knee osteoarthritis. In a previous systematic review of the literature, six main phenotypes were identified: Minimal Joint Disease (MJD), Malaligned Biomechanical (MB), Chronic Pain (CP), Inflammatory (I), Metabolic Syndrome (MS) and Bone and Cartilage Metabolism (BCM). The purpose of this study was to classify a sample of individuals with knee osteoarthritis (KOA) into pre-defined groups characterized by specific variables that can be linked to different disease mechanisms, and compare these phenotypes for demographic and health outcomes.

**Methods:**

599 patients were selected from the OAI database FNIH at 24 months’ time to conduct the study. For each phenotype, cut offs of key variables were identified matching the results from previous studies in the field and the data available for the sample. The selection process consisted of 3 steps. At the end of each step, the subjects classified were excluded from the further classification stages. Patients meeting the criteria for more than one phenotype were classified separately into a ‘complex KOA’ group.

**Results:**

Phenotype allocation (including complex KOA) was successful for 84% of cases with an overlap of 20%. Disease duration was shorter in the MJD while the CP phenotype included a larger number of Women (81%). A significant effect of phenotypes on WOMAC pain (F = 16.736 p <0.001) and WOMAC physical function (F = 14.676, p < 0.001) was identified after controlling for disease duration.

**Conclusion:**

This study signifies the feasibility of a classification of KOA subjects in distinct phenotypes based on subgroup-specific characteristics.

## 1. Introduction

Osteoarthritis (OA) is the most common form of arthritis and the knee is the most commonly affected joint [1]. The knee osteoarthritis (KOA) patient population is highly heterogeneous. To better explain this heterogeneity it has been suggested that a multitude of underlying mechanisms leading to similar clinical presentations (joint damage, pain, stiffness and loss of physical function) are responsible for the development and progression of KOA[2,3]; in other words, the KOA population comprises a number of distinct subgroups, or phenotypes[2,4,5].

Different approaches to phenotype identification in the KOA population have been advocated. Felson et al. suggested that phenotype research should be focused on those subgroups that could influence treatment allocation and management of the disease[5]. Several authors have attempted to identify meaningful clinical phenotypes of the disease[6] A common approach emerging from the literature is to use risk or etiologic factors (e.g. obesity, skeletal malalignment, depression) to identify groups characterized by major hypothesised underlying mechanisms (e.g. pain sensitisation, excessive joint load, metabolic changes).

A recent systematic review summarized these studies[6], suggesting the existence of specific sets of variables that describe subgroups characterised by: unusual inflammation profiles inside the knee joint; sensitisation to pain and pain neurophysiology alterations (e.g. generalized hypersensitivity to pain and temporal summation); adverse biomechanics where the knee malalignment is severe enough to drive the disease in absence of other known risk factors; systemic metabolic disorders (e.g. obesity, diabetes); and changes in bone and cartilage metabolism in the knee, as well as a phenotype of minimal joint disease with minor symptoms and discomfort over an extensive period of time.

A next essential step in the development of a feasible and useful phenotypic sub-classification of KOA is to establish whether individual patients can be correctly assigned to these phenotypes. Assigning a phenotype should then enable better clinical decision making with respect to optimal treatment strategies[7–9]. Additionally, the phenotypes previously identified are not necessarily mutually exclusive, i.e. the key characteristics of more than one phenotype could be present in the same patient. This raises the question whether this group of poly-phenotypical patients, or ‘complex KOA’ cases, is characterised by a more severe disease status and course.

The purpose of this study was to classify a sample of individuals with KOA into pre-defined groups characterized by specific characteristics that can be linked to different disease mechanisms and/or targeted by tailored treatments (e.g. subjects with malalignment only, in absence of other risk factors, may be considered belonging to a malaligned biomechanical phenotype)

Secondly, these groups of patients were then compared for demographic and health outcomes (Western Ontario and McMaster Universities Osteoarthritis Index [WOMAC] pain, WOMAC physical function) to identify differences in severity and prognosis between phenotypes. Of particular interest was the group of ‘complex KOA’ patients who met the criteria for more than one phenotype.

## 2. Methods

### 2.1 Study population

Data were obtained from the Osteoarthritis Initiative (OAI) database, a multi-centre prospective longitudinal cohort study designed to identify biomarkers and risk factors associated with the incidence and progression of KOA (http://www.oai.ucsf.edu/)[10]. As part of the OAI, participants aged 45–79 years with, or at risk of, symptomatic KOA in at least one knee were recruited from four centres in the United States.

Data from the OA Biomarkers Consortium FNIH Project was used as the sample source for the current study. The FNIH study includes 600 subjects from the OAI dataset with availability at baseline and 24 months of knee radiographs, knee magnetic resonance images (MRI), stored serum and urine specimens and clinical data. Knees were not included in the FNIH cohort if they received a joint replacement before the 24 months follow up, had a minimum medial joint space width <1.0mm and/or WOMAC pain >91 on 0–100 scale. For the current study, the 24 month follow up was selected for the larger availability of data. We selected OAI participants who had: (1) Kellgren & Lawrence grade (K&L) ≥1; (2) availability of Magnetic Resonance Imaging Osteoarthritis Knee Score (MOAKS) score data; and (3) serum and urine biomarkers of bone and cartilage metabolism. MRI assessment in the selected cohort was performed unilaterally; therefore the index knee was the one with the availability of MOAKS score[11].

### 2.2 Selection of the variables for the classification process

Six main sets of variables suggesting the existence of clinical KOA phenotypes were identified through a previous search of the literature[6] (Table 1). In order to design the classification process, cut-off values were identified for each of these sets of variables and used to determine the phenotype membership of each subject. Where possible, validated cut-offs already used in OA research or clinical practice were adopted (e.g. Centre for Epidemiologic Studies Depression scale [CES-D], malalignment). Where existing cut-offs were not available, specific values were determined based on variables distribution in the selected sample (Table 1). For a detailed description of the variables see S1 Table.

**Table 1:** Criteria used in the classification process.

Phenotype	Specific cut-offs	Sensitive cut-offs
MJD	(Pain* ≤ 3 AND K&L ≤ 2 at 24 months) AND (Pain ≤ 3 AND K&L ≤ 2 at 48 months).	-
I	MOAKS score synovitis/effusion = 3	MOAKS score synovitis/effusion = 2
MD	Presence of diabetes AND BMI ≥ 30	(Presence of diabetes OR BMI ≥ 30) AND (systolic pressure ≥ 140 mmHg OR diastolic pressure ≥ 100 mmHg)
CP	CESD-R ≥ 16 OR Tender points n° ≥ 6 located: above and below the waist, on both sides of the body, and axially	CESD-R ≥ 16 OR Tender points n° ≥ 3 located above and below the waist
MB	Valgus alignment ≥2° AND MOAKS lateral tibial condyle ≥ 2.0 AND MOAKS medial tibial condyle ≤1.0) OR (Varus alignment ≥2° AND MOAKS lateral tibial condyle ≤ 1.0 AND MOAKS medial tibial condyle ≥ 2.0)	Valgus alignment ≥2° AND MOAKS lateral tibial condyle > MOAKS score medial tibial condyle) OR (Varus alignment ≥2° AND MOAKS lateral tibial condyle < MOAKS score medial tibial condyle)
BCM	(uNTXI, uCTXII, uCTX-1ß, uCTX-1α > 75^th^ percentile) OR (sComp, sHA >75^th^ percentile)	(uNTXI, uCTXII, uCTX-1ß, uCTX-1α > 65^th^ percentile) OR (sComp, sHA >65^th^ percentile)

*Worse pain in the past seven days rated from 0–10

K&L: Kellgren & Lawrence

MOAKS: Magnetic Resonance Imaging Osteoarthritis Knee Score

MJD: minimal Joint Disease phenotype

I: Inflammatory phenotype

MD: Metabolic Disorder phenotype

CP: Chronic Pain phenotype

MB: Malaligned Biomechanical phenotype

BCM: Bone and Cartilage Metabolism phenotype

BMI: body mass index

CES-D: Center for Epidemiologic Studies Depression scale

uNTXI: Urine N-Terminal Telopeptide type I

uCTXII: C-Terminal Telopeptide type II

uCTX-1ß: C-Terminal Telopeptide type 1ß

uCTX-1α: C-Terminal Telopeptide type 1α

sComp: Serum Cartilage Oligomeric Matrix Protein

sHA: Serum Hyaluronic Acid

### 2.3 Classification process

**Fig 1:**
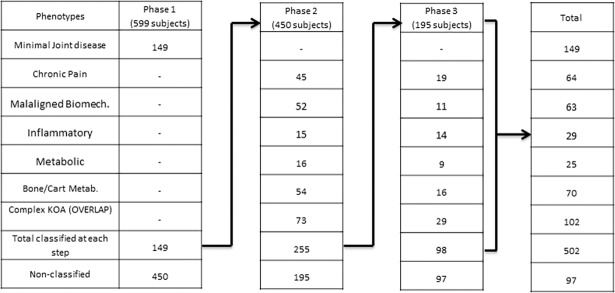
Summary of the subjects classified in each classification phase.

The classification process consisted of three stages. For an overview of the classification process see Fig 1. In the first stage, the Minimal Joint Disease (MJD) phenotype was identified starting from the whole sample. Subjects included in the MJD phenotype represent a subgroup with low to mild symptomatology which is stable over time and is associated with minor health care needs[5]. For these reasons, we chose to classify subjects in the MJD phenotype in the first stage and exclude them from the following classification steps. In the second stage, cut-offs were designed to classify subjects in the remaining five phenotypes. The cut-offs used at this stage were characterized by high specificity in order to minimize false positive results. In the third step, the cut-offs previously applied were modified to increase their sensitivity in order to allocate the subjects not yet classified after the first two steps. This last step was included to minimize false negative results in subjects not classified in the previous step due to the high specificity of the criteria. The risk of false positives at this stage was reduced since the sensitive criteria were applied only to the subjects not classified in the previous steps. Any remaining patient not allocated to a phenotype after the third step was regarded as not fitting in any of the phenotypic subgroups (‘NON-CLASSIFIED’ group). In the second and third steps, the subjects who met the criteria for more than one phenotype were allowed to overlap and were grouped together in a separate group named ‘complex KOA’. This three-step design allowed us to classify the highest possible number of subjects limiting the excessive overlap that would emerge from the use of only sensitive criteria and avoiding the classification of subjects in small size groups that would result from the use of only specific criteria.

### 2.4 Minimal joint disease phenotype (MJD)

A combination of low level of self-reported pain (worse pain in the last week ≤ 3/10; release version 3.2.1) and mild to moderate radiographic OA (ROA) (K&L ≤ 2, release version 3.7) was chosen to classify subjects in the MJD phenotype (see Table 1). To ensure the inclusion of subjects with minimal/no disease progression, exclusively for the MJD, the aforementioned cut-offs were applied at both the 24- and 48- month OAI follow-up measurements (K&L release version 6.5, Self-reported pain release version 6.2.1). Eight subjects satisfying the inclusion criteria at 24 month had K/L grade missing at 48 month. Only for these subjects, the following criteria were used to avoid misclassifications: (Worst self-reported pain in the last week ≤1 on a scale 0–10 at 24 months) AND (K&L score ≤2 at t0) AND (Worst self-reported pain in the last week ≤1 on a scale 0–10 at 48 months).

### 2.5 Chronic pain phenotype(CP)

Depression is a common condition among subjects with a chronic painful disease such as KOA where it has an important interaction with pain perception and functional decline[12]. Widespread pain represents an important indicator of enhanced pain sensitivity and central sensitization and is associated with high level of pain in the KOA population[13]. A combination of depression and self-reported widespread pain (release 3.2.1) was used to classify patients belonging to the chronic pain phenotype. A score of 16 in the CES-D was used as a cut-off for depression in both specific and sensitive criteria since it has been associated with clinically significant depressive symptoms[14]. Ten body areas (excluding the knees) were identified to cover the areas used in the ACR criteria (1990) for widespread pain (Neck, left shoulder, right shoulder, temporomandibular joint (jaw) either side, temporomandibular joint across face/cheek, left elbow, right elbow, lower back, left hip, right hip). The presence of depression and pain in at least 6 body spot located: above and below the waist, on both sides of the body, and axially was used as cut-offs for the identification of widespread pain in the specific criterion. Presence of depression and 3 painful body areas located above and below the waist was used as a sensitive criterion.

### 2.6 Malaligned biomechanical phenotype (MB)

Repeated evidence shows that knees sufficiently malaligned progress to worse disease without the presence of other known risk factors[5]. Malalignment confers high focal stress that can overcome the ability of cartilage within the joint to withstand it. Therefore, compartmental degeneration consistent with the alignment of the lower limb may identify a KOA phenotype in which biomechanical mechanisms are responsible for the disease[15,16]. For this reason, malalignment and cartilage degeneration were the variables used to classify subjects in the malaligned biomechanical phenotype. A variation of ±2° from the hip knee ankle angle, assessed clinically with hand goniometer (release 3.2.1), was considered as malalignment, in both the specific (step 2) and sensitive (step 3) criteria[17]. Cartilage degeneration was assessed using the MOAKS score (Femoral compartment: cartilage area intra-rater reliability kappa = 0.92, inter-rater reliability kappa = 0.62; tibial compartment: cartilage area intra-rater reliability kappa = 0.73, inter-rater reliability kappa = 0.36) (release 3.1). For the purpose of this study, only the central weight-bearing part of the tibial condyles was analysed since it showed to be the most affected by OA degeneration (in the selected sample). The aforementioned cut-offs were combined in order to develop the specific and sensitive criteria. In the specific criteria the MOAKS score for the most involved compartment was fixed at 2.0 (indicating damage that involves between 10% and 75% of the cartilage surface without full-thickness lesion) or higher while the less affected was fixed at 1.0 or lower (indicating damage that involves less than 10% of the cartilage surface without full-thickness lesion). The most degenerated compartment had to be consistent with malalignment (e.g., higher damage in the medial compartment with varus malalignment). In the sensitive criteria, no specific value for the MOAKS score was selected, the only condition was that the compartment with the highest level of degeneration was consistent with the malalignment.

### 2.7 Inflammatory phenotype (I)

Synovitis is a strong indicator of the presence of inflammation processes and has been reported as a key factor in an inflammatory phenotype[18]. The presence of synovitis was graded using the MOAKS score (release 3.1) for synovitis/effusion on a scale from 0 (no effusion/synovitis) to 3 (severe effusion/synovitis). Subjects with a MOAKS synovitis/effusion score of 3 (specific criterion, step 2) and 2 (sensitive criterion, step 3) were included in the inflammatory phenotype.

### 2.8 Metabolic disorders phenotype (MD)

Previous studies showed the existence of a phenotype in which the individuals are characterized by the presence of metabolic disorders (e.g. Obesity, diabetes, hypertension, dyslipidemia)[18–22]. High BMI (≥30kg.m^-2^) and the presence of diabetes are two of the most common metabolic disorders and have been shown to be related to a higher incidence of KOA and a higher risk of knee replacement[23]. Therefore, in the present study, people with diabetes and BMI ≥30 kg/m^2^ were considered to belong to a metabolic disorder phenotype (MD). High blood pressure is another recognized disorder part of the metabolic syndrome. However, it has been shown to have only a weak association with KOA[24,25]. Recent studies suggested that hypertension should only be considered an aggravating factor for OA in subjects with obesity or other metabolic disorders[26]. For this reason, in order to increase the sensitivity of the criterion, any one of the previous specific criteria in combination with high blood pressure/hypertension (systolic pressure ≥ 140 mmHg OR diastolic pressure ≥ 100 mmHg) was considered sufficient to include a subject in the MD phenotype in the last, sensitive, step of the classification process.

### 2.9 Bone and cartilage metabolism (BCM)

Alterations in bone and cartilage metabolism have been identified in specific KOA subgroups[18,27–29]. Biomarker analysis is the gold standard for the identification of metabolic alterations of bone and cartilage. Van Spil et al. identified two subgroups of KOA subjects characterised by an alteration in the bone and cartilage metabolism through a principal component analysis [18]. The biomarkers available in the OAI dataset matching with the biomarkers characterizing the two groups identified by van Spil et al. were extracted for the purpose of this study (1: uNTXI, uCTXII, uCTX-1ß, uCTX-1α; 2: sComp, sHA) (release version 3.2.1). A concentration higher than the 75^th^ percentile of each of the biomarkers of at least one of the subgroups was used as a specific cut-off to determine the membership to the bone and cartilage metabolic phenotype in the first selection step. The cut-off was lowered to the 65^th^ percentile in the second step to increase its sensitivity.

### 2.10 Comparison of phenotypes for demographic and health outcomes

Differences in sex and additional variables including history of knee injury (defined as any self-reported knee injury severe enough to limit the ability to walk for at least two days previous the 24 month visit), knee joint laxity and quadriceps strength were analysed in order to better define the phenotypes. One variable for knee pain (WOMAC pain 24 month) [30] and one variable for activity limitation (WOMAC function 24 month) [30] were used to assess differences between the groups and regarded as a health outcome. A difference between groups of 9 points on a 0–100 scale for WOMAC pain was considered clinically significant[31].

### 2.12 Analysis

Subjects included in the same phenotype but in different steps of the classification process were grouped and analysed as a single phenotype. Subjects classified in more than one phenotype were considered part of the complex KOA subgroup and analysed as a separate subgroup. Subjects not classified in any of the phenotypes were considered and analysed as a separate subgroup. A One-Way ANOVA with Bonferroni correction was conducted to analyse the difference in age and disease duration between the groups. A One-way ANCOVA was conducted to determine whether a statistically significant difference existed between phenotypes on the selected clinical variables (i.e. WOMAC pain, WOMAC physical function, muscle strength) controlling for disease duration and use of pain medications for the knee symptoms. A post hoc Sidak test was used to correct for multiple comparisons; p values and confidence intervals were bootstrapped (bias-corrected accelerated 1000 samples). A Chi-square test was conducted to test the relation between the categorical variables (e.g. gender, history of knee injury) and phenotypes. Fisher’s exact test was used for the K&L score, laxity and synovitis. The cell counts were compared across phenotypes using a z-test and a Bonferroni correction for multiple comparisons (significance value of p < 0.05). All the calculations were performed using IBM SPSS statistics v22.

## 3. Results

For the purpose of the study, 599 subjects were selected (characteristics of the sample reported in Table 2 and supplementary material 1A). Therefore, from the original sample of the FNIH sub-cohort, only one subject presenting K&L grade <1 was excluded. In the first step of the selection process, 149 of the 599 subjects were classified into the MJD phenotype. In the second step, 255 of the 450 remaining subjects were classified in the other five phenotypes with an overlap of 28.6% (73/255; 61 classified in 2 phenotypes, 12 classified in 3 phenotypes). In the third step, 98 of the 195 remaining subjects were classified using the high sensitivity criteria with an overlap of 29.6% (29/98, 24 classified in two phenotypes, five classified in three phenotypes). At the end of the process, 502 subjects (83%) were classified in at least one phenotype with a total overlap of 20% (102 out of 502). Ninety-seven subjects (17%) were not classified in any phenotype at the end of the process (see Table 2). No specific pattern was identified in the overlap among the phenotypes (Table 3).

**Table 2:** Sample characteristics.

Variable	N	Mean	SD
Age	599	63.6	8.8
Disease duration (years)	599	3.6	2
Sex (female)	353		
Alignment	599		
Varus	228	38.7%	
Valgus	184	31.2%	
K&L score	599		
1		8%	
2		48.4%	
3		40.4%	
4		3%	
Laxity	529		
No laxity		60.3%	
Mild		34.4%	
Moderate/severe		5.3%	
Worse pain previous week	592	3	2.8
WOMAC synovitis/effusion			
Serum Comp (ng/mL)	599	786.6	318.7
Serum HA (ng/mL)	599	67.9	64.6
Urine NTXI (nmol BCE)	599	253.2	192.0
Urine CTXII (ug/L)	599	2.6	2.3
Urine CTX-1a (ng/mL)	599	3.6	2.4
Urine CTX-1ß (ug/L)	599	17.4	15.8
BMI (kg.m^-2^)	597	30.7	4.8
Blood pressure: systolic (mm Hg)	597	124.6	15.3
Blood pressure: diastolic (mm Hg)	597	75.47	9.5
CES-D	592	6.82	6.8
Widespread pain	597	1.8	1.7
Quadriceps Strength	563	324.5	126.9
WOMAC physical function	599	9.5	11.3
WOMAC pain	599	3	3.5

BMI: body mass index

CES-D: Center for Epidemiologic Studies Depression scale

uNTXI: Urine N-Terminal Telopeptide type I

uCTXII: C-Terminal Telopeptide type II

uCTX-1ß: C-Terminal Telopeptide type 1ß

uCTX-1α: C-Terminal Telopeptide type 1α

sComp: Serum Cartilage Oligomeric Matrix Protein

sHA: Serum Hyaluronic Acid

nM BCE: nanomoles bone collagen equivalents

**Table 3:** Report of the overlap between phenotypes.

	MJD (1)	CP (2)	MB (3)	I (4)	MD (5)	BCM (6)
MJD (1)	-	-	-	-	-	-
CP (2)		-	9	5	14	17
MB (3)			-	4	5	10
I (4)				-	1	13
MD (5)					-	7
BCM (6)						-

Subjects classified in three phenotypes: I+BCM+CP: 4, I+CP+ MD: 2, BCM+CP+MB: 6, MD+CP+MB: 3, BCM+MD+MB: 1, BCM+MD+CP: 1; Total: 17

MJD: minimal Joint Disease phenotype

I: Inflammatory phenotype

MD: Metabolic Disorder phenotype

CP: Chronic Pain phenotype

MB: Malaligned Biomechanical phenotype

BCM: Bone and Cartilage Metabolism phenotype

No difference in mean age was identified (Table 4). The MJD phenotype showed a shorter symptoms duration (p = 0.001) compared to the CP phenotype and the complex KOA subgroup (P = 0.018), while no statistical difference was detected between the other phenotypes. Sex was not equally distributed between all the phenotypes. The prevalence of women reached 81% in the CP phenotype while it was only 35% in the MB phenotype (p <0.05). No differences regarding sex distribution were found between the other groups.

**Table 4:** Comparison of patient characteristics between groups.

PHENOTYPE	MJD (1)	CP (2)	MB (3)	I (4)	MD (5)	BCM (6)	COMPLEX KOA (7)	NON CLASS (8)
	Mean (SD)	Mean (SD)	Mean (SD)	Mean (SD)	Mean (SD)	Mean (SD)	Mean (SD)	Mean (SD)
Number	149	64	63	29	25	70	102	97
Age	63.1 (8.8)	63.4 (8.1)	65.6 (8.5)	63.0 (9.6)	61.7 (8.7)	65.3 (8.5)	63.5 (9.4)	62.9 (8.9)
Gender (female)	58%^2^^,^^3^	81%^1^^,^^3^^,^^6^	35%^1^^,^^2^^,^^7^	62%	60%	54%^2^	63%^2^	59%
Disease duration (years)	3^2^^,^^7^ (1.7)	4.4^1^ (2.2)	3.6 (2)	3.8 (2)	3.5 (1.8)	3.7 (2.0)	3.9^1^ (2.1)	3.8 (2.0)
History of injuries	29.5%	46.9%	36.5%	44.8%	36.0%	35.7%	36.7%	39.2%
WOMAC physical function (0–100)†	7.0^all^ (1.2)	20.3^1^^,^^3^^,^^6^^,^^8^ (1.8)	11.0^1^^,^^2^^,^^7^^.^ (1.8)	15.9^1^^,^^7^ (2.6)	18.0^1^ (2.8)	13.6^1^^,^^2^^,^^7^^.^ (1.6)	24.0^1^^,^^3^^,^^4^^,^^6^^,^^8^(1.4)	10.7^1^^,^^2^^,^^6^^,^^7^ (1.4)
WOMAC Pain (0–100)†	6.6^all^ (1.2)	19.5^1^^,^^3^^,^^7^^,^^8^ (1.9)	13.0^1^^,^^2^^,^^7^ (1.8)	18.1^1^^,^^7^ (2.7)	18.4^1^^,^^7^ (2.9)	15.6^1^^,^^7^ (1.8)	26.8^all^ (1.5)	12.1^1^^,^^2^^,^^4^^,^^7^ (1.5)

All: significantly different from all the other phenotypes (p < 0,05)

1: significantly different from phenotype 1 (MJD) (p < 0,05)

2: significantly different from phenotype 2 (CP) (p < 0,05)

3: significantly different from phenotype 3 (MB) (p < 0,05)

4: significantly different from phenotype 4 (I) (p < 0,05)

5: significantly different from phenotype 5 (MS) (p < 0,05)

6: significantly different from phenotype 6 (BCM) (p < 0,05)

7: Significantly different from group 7 (COMPLEX KOA) (p < 0,05)

8: Significantly different from group 8 (NON-CLASS.) (p < 0,05)

MJD: minimal Joint Disease phenotype

I: Inflammatory phenotype

MD: Metabolic disorder phenotype

CP: Chronic Pain phenotype

MB: Malaligned Biomechanical phenotype

BCM: Bone and Cartilage Metabolism phenotype

†: as result of the ANCOVA with disease duration and pain medication use as covariate the value reported represent the adjusted mean and standard error.

There was a significant effect of phenotypes on WOMAC pain (F = 16.736 p = 0.001) and WOMAC physical function (F = 14.674, p = 0.001) after controlling for disease duration and use of pain medication (Table 5). The assumption of homogeneity of the regression slope was not violated. Looking at the health outcomes, the MJD phenotype showed the lowest scores of WOMAC pain and WOMAC physical function (p<0.05 compared to all other groups). The CP phenotype presented the highest pain severity and lowest WOMAC physical function score when compared with the other individual phenotypes (WOMAC pain p = 0.001 compared to MJD and MB; WOMAC physical function p = 0.001 compared to MJD and MB, and p = 0.03 compared to BCM). WOMAC pain score differences were also clinically significant. The MB phenotype presents the second lowest score (after the MJD phenotype) for WOMAC pain and physical function compared to the other phenotypes (WOMAC pain p = 0.001 compared to MJD, CP; WOMAC physical function p = 0.001 compared to MJD, CP). The difference in pain between the MB and CP phenotype was above the threshold for clinical significance. The I and MD phenotypes had similar scores in all the WOMAC areas (no significant difference) while they had intermediate scores if compared with the other phenotypes. The BCM phenotype had lower scores compared to the CP, I and MD phenotypes in all the WOMAC areas; however, only the difference in WOMAC function with the CP phenotype was significant (p = 0.03).

**Table 5:** Ancova models statistics.

	WOMAC PAIN			WOMAC PHYSICAL FUNCTION		
Source	Type III Sum of Squares	F	Sig.	Type III Sum of Squares	F	Sig.
Corrected Model	53995.769^a^	27.700	<0.001	48931.351^a^	27.677	<0.001
Intercept	6447.274	29.768	<0.001	4274.415	21.759	<0.001
Medication use	10216.744	47.171	<0.001	2073.871	10.557	<0.001
Disease duration	1893.982	8.745	0.003	10950.318	55.743	0.001
Phenotype	25373.689	16.736	<0.001	20178.351	14.674	<0.001
Error	127353.416			115507.483		
Total	317856.250			281992.661		
Corrected Total	181349.185			164438.834		

a: R Squared = .298 (Adjusted R Squared = .290)

b: R Squared = .298 (Adjusted R Squared = .287)

df: degree of freedom

The complex KOA subgroup showed the worst score in all the WOMAC areas compared to the other groups (WOMAC pain p < 0.05 compared to all other groups, the difference was clinically significant when compared with MJD, MB, BCM, non-classified). In contrast, the group composed of non-classified subjects showed the second lowest WOMAC scores among all subgroups. The non-classified subgroup showed a mild disease (low levels of pain and disability) and moderate radiographic degeneration (KL grade 2: 36.1%; grade 3: 55.7%).

Regarding the alignment, the MB phenotype is composed mainly of subjects with varus malalignment. In the other subgroups, varus and valgus malalignment had a similar distribution. The CP phenotype showed the lowest quadriceps strength while the MB showed the highest quadriceps strength (Table 6).

**Table 6:** Summary of variables used to classify the subjects in to the hypothesized phenotypes.

PHENOTYPE	MJD (1)	CP (2)	MB (3)	I (4)	MD (5)	BCM (6)	COMPLEX KOA (7)	NON CLASS (8)
	Mean (SD)	Mean (SD)	Mean (SD)	Mean (SD)	Mean (SD)	Mean (SD)	Mean (SD)	Mean (SD)
Number	149	64	63	29	25	70	102	97
Alignment								
Neutral	29.0%^3^	41.9%^3^	0.0%^all^	34.5%^3^	28.0%^3^	40.6%^3^	20.8%^3^^,^^8^	45.3 ^3^^,^^7^%
Varus	32.4%^3^	24.2%^3^^,^^7^	79.4%^all^	27.6%^3^	40.0%^3^	30.4%^3^	50.5%^2^^,^^3^^,^^8^	27.4%^3^^,^^7^
Valgus	38.6%	33.9%	20.6%	37.9%	32.0%	29.0%	28.7%	27.4%
K&L score								
1	14.8%^7^	6.3%	9.5%	6.9%	8.0%	5.7%	2.0%	7.2%
2	85.2%^all^	43.8%^1^	23.8%^1^	41.4%^1^	56.0%^1^	35.7%^1^	33.3%^1^	36.1%^1^
3	0.0%^all^	48.4%^1^	60.3%^1^	44.8%^1^	32.0%^1^	54.3%^1^	58.8%^1^	55.7%^1^
4	0.0%^4^	1.6%	6.3%	6.9%^1^	4.0%	4.3%	5.9%	1.0%
Worse pain previous week	0.7^all^ (1)	4.2 ^1^^,^^8^ (2.6)	3.1^1^^,^^7^ (2.4)	3.8^1^ (2.6)	4.1^1^ (2.8)	3.5^1^ (3)	4.8^1^^,^^3^^,^^8^ (3)	2.7^1^^,^^2^^,^^7^ (2.5)
MOAKS synovitis/effusion								
None	38.1^4^%	32.3^4^%	31.7^4^%	0.0^1^^,^^2^^,^^3^^,^^5^^,^^8^%	33.3^4^%	23.1^8^%	23.4^8^%	52.8^4^^,6,^^7^%
moderate	45.3%^4^	56.5%^4^	45.0%^4^	0.0%^all^	45.8%^4^	53.8%^4^	33.0%^4^	47.2%^4^
Severe (MOAKS score 2–3)	16.5%^4^^,^^7^^,^^8^	11.3%^4^^,^^7^^,^^8^	23.3%^4^^,^^7^^,^^8^	100%^all^	20.8%^4^^,^^8^	23.1%^4^^,^^7^^,^^8^	43.6%^4^^,^^8^	0.0%^all^
Serum Comp, ng/mL	780.7 (341.9)	723^6^^,^^7^ (222.5)	715.8^6^^,^^7^ (231.9)	759.6 (361)	742.5 (246.6)	908.5^2^^,^^3^^,^^5^^,^^8^ (324)	882.5^2^^,^^3^^,^^8^ (395.1)	726.5^6^^,^^7^ (256)
Serum HA, ng/mL	61.4^6^ (31.5)	57.6^6^ (18.4)	58.7^6^ (25.4)	60.7^6^ (25.5)	58.2^6^ (18.8)	88.0^1^^,^^2^^,^^3^^,^^4^^,^^5^^,^^8^ (55.9)	92.5 (136.9)	57.3^6^ (22.5)
Urine NTXI, nmol BCE	232.4^6^ (158.6)	191.6^6^ (128.4)	206.6^6^ (114.2)	181.4^6^ (103.4)	249.3^6^ (113)	442^all^ (293.6)	302.4^6^ (236.8)	204.4^6^ (123.4)
Urine CTXII, ug/L	2.1^5^^,^^6^^,^^7^ (1.8)	2^5^^,^^6^^,^^7^ (1.7)	2.2^5^^,^^6^^,^^7^ (1.3)	2^5^^,^^6^^,^^7^ (1.6)	3.1^1^^,^^2^^,^^3^^,^^4^^,^^6^^,^^8^ (1.8)	4.5^1^^,^^2^^,^^3^^,^^4^^,^^5^^,^^8^ (3.1)	3.6^1^^,^^2^^,^^3^^,^^4^^,^^8^ (3.3)	1.8^5^^,^^6^^,^^7^ (1.2)
Urine CTX-1a, ng/mL	3.3 ^4^^,^^6^^,^^7^ (1.6)	3.1^6^^,^^7^ (1.4)	3.0^6^^,^^7^ (0.9)	2.8 ^1^^,^^5^^,^^6^^,^^7^ (0.7)	3.2^4^^,^^6^^,^^7^ (1.1)	6.2^all^ (4.5)	4.2^1^^,^^2^^,^^3^^,^^4^^,^^6^^,^^8^ (2.8)	3.2^6^^,^^7^ (1.4)
Urine CTX-1ß, ug/L	15.9 ^4^^,^^6^^,^^7^^,^^8^ (13)	13.6^6^^,^^7^ (10.4)	13.3^6^^,^^7^ (9.5)	11.6 ^1^^,^^5^^,^^6^^,^^7^ (9.2)	16.8^4^^,^^6^ (9.5)	32.8^all^ (25.7)	21.0^1^^,^^2^^,^^3^^,^^4^^,^^8^ (19.3)	13.2^4^^,^^6^^,^^7^ (8.7)
BMI(kg.m^-2^)	30.2 ^5^^,^^6^^,^^7^ (4.4)	30.6 ^7^ (5.2)	29.4^5^^,^^6^^,^^7^ (4)	31.0 (5.2)	33.5^1^^,^^3^^,^^8^ (4.5)	31.8^1^^,^^3^^,^^8^ (5.2)	32.4^1^^,^^3^^,^^8^ (5)	29.5^5^^,^^6^^,^^7^ (4.6)
Diabetes	6.7%^5^^,^^7^	1.6%^5^^,^^7^	3.2%^5^^,^^7^	6.9%^5^^,^^7^	68.0^1^^,^^2^^,^^3^^,^^4^^,^^6^^,^^8^%	1.4%^5^^,^^7^	26.5% ^1^^,^^2^^,^^3^^,^^4^^,^^6^^,^^8^	1.0%^5^^,^^7^
Blood pressure: systolic, mm Hg	123.3 (15.6)	125.0 (16.8)	123.5 (14.3)	121.9 (10.2)	122.2 (13.9)	127.2 (16)	128 (16.3)	123.2 (14)
Blood pressure: diastolic, mm Hg	75.7 (8.9)	76.3 (10.3)	74.9 (9.8)	75.8 (10.1)	74.1 (8.7)	76 (8.9)	75.3 (11.4)	75.2 (8.5)
CES-D	5.7 ^2^^,^^7^ (5.9)	12.5 ^1^^,^^3^^,^^4^^,^^5^^,^^6^^,^^8^ (8.3)	4.3 ^2^^,^^7^ (3.4)	4.3 ^2^^,^^7^ (3.4)	6.5 ^2^^,^^7^ (5.5)	5.1 ^2^^,^^7^ (4.4)	10.9 ^1^^,^^3^^,^^4^^,^^5^^,^^6^^,^^8^ (9)	3.9 ^2^^,^^7^(3.8)
Widespread pain	1.32^2^^,^^7^ (1.3)	3.8^all^ (2)	1.2^2^^,^^7^ (1.1)	1.1^2^^,^^7^ (1.1	1.4^2^^,^^7^ (1.1)	1.3^2^^,^^7^ (1.1)	2.7^all^ (1.9)	1.0^2^^,^^7^ (0.8)
Quadriceps Strength, N/kg	4.0^2^ (1.3)	3.3^1^^,^^3^^,^^8^ (1.4)	4.2^2^^,^^7^ (1.4)	3.3 (0.9)	3.3 (1.3)	3.7 (1.3)	3.4^3^^,^^8^ (1.4)	4^2^^,^^7^ (137)

All: significantly different from all the other phenotypes (p < 0,05)

1: significantly different from phenotype 1 (MJD) (p < 0,05)

2: significantly different from phenotype 2 (CP) (p < 0,05)

3: significantly different from phenotype 3 (MB) (p < 0,05)

4: significantly different from phenotype 4 (I) (p < 0,05)

5: significantly different from phenotype 5 (MS) (p < 0,05)

6: significantly different from phenotype 6 (BCM) (p < 0,05)

7: Significantly different from group 7 (COMPLEX KOA) (p < 0,05)

8: Significantly different from group 8 (NON-CLASS.) (p < 0,05)

MJD: minimal Joint Disease phenotype

I: Inflammatory phenotype

MD: Metabolic Disorder phenotype

CP: Chronic Pain phenotype

MB: Malaligned Biomechanical phenotype

BCM: Bone and Cartilage Metabolism phenotype

BMI: body mass index

CES-D: Center for Epidemiologic Studies Depression scale

uNTXI: Urine N-Terminal Telopeptide type I

uCTXII: C-Terminal Telopeptide type II

uCTX-1ß: C-Terminal Telopeptide type 1ß

uCTX-1α: C-Terminal Telopeptide type 1α

sComp: Serum Cartilage Oligomeric Matrix Protein

sHA: Serum Hyaluronic Acid

nM BCE: nanomoles bone collagen equivalents

## 4. Discussion

In the present study patients with KOA were classified into six phenotypes using pre-determined criteria, with patients meeting the criteria for more than one phenotype classified separately into a ‘complex KOA’ group. Phenotype allocation (including complex KOA) was successful for 84.1% of cases using characteristics that can be measured in a clinical setting.

Subjects classified in the MJD group showed shorter disease duration compared to the other phenotypes (difference with other groups varying from 0.6 to 1.4 years). This could suggest that these subjects constitute an early KOA subgroup and for this reason have a lower symptomatology compared with the other phenotypes. Following this hypothesis, the subjects included in the MJD phenotype should progress and reach (or even overcome) the level of symptoms showed by the other phenotype in two years.

However, subjects classified in the MJD were selected to have minimal or no progression of symptoms at two years follow-up. This means that the MJD subjects, despite an increase of two years in the disease duration, had minimal or no change in symptoms severity. Moreover, it must be considered that the MJD showed a low prevalence of comorbidities (e.g. depression, diabetes) (Table 6) that combined with the slow radiographic progression confirms the finding of previous longitudinal studies that attempted to identify minimal joint disease phenotypes[32]. Despite the fact that it is not yet clear whether a specific disease mechanism is responsible for the slow progression rate and mild symptoms that characterize this group, the identification of subjects who do not experience symptomatic worsening would be critical for the improvement of resource and treatment allocation which may ultimately result in a reduction of the clinical cost for KOA.

Subjects classified in the CP phenotype demonstrated the highest levels of pain, disability and the lowest muscle strength. In this phenotype, the coexistence of cognitive mechanisms and neurophysiologic alterations such as widespread pain suggests a more global disease mechanism in which the central nervous system has a primary role and therefore could represent a target for specific treatments. Carlesso et al., in a study published in 2016[33], suggested that chronic pain related symptoms are consequences of the knee disease while Neogi et al. suggested that sensitization and hypersensitivity [34], as well as depression, might be present before KOA. Despite the contrasting evidence regarding the causality relationship between KOA and central pain mechanisms; once chronic pain is present, a cognitive-behavioural intervention and pain education may be worthwhile and may optimize the results of other traditional intervention such as exercise therapy and joint replacement[35].

The MB phenotype might be a subtype of active subjects, with high levels of muscle strength, high prevalence of malalignment, lower BMI and low prevalence of other comorbidities. Furthermore, despite the severe radiographic degeneration present in this phenotype (60% of the subjects had a K&L grade ≥ 3); subjects classified in the MB phenotype have low levels of pain and disability. These findings suggest the existence of a “healthy” (low levels of comorbidity, depression, widespread pain) subtype of OA subjects in which biomechanical factors seem to be the main disease mechanisms. Similar criteria to the specific ones used in the current study to classify subjects in the MB have been applied in an external cohort to subjects with varus malalignment. The results showed that subjects classified using these criteria were linked to increased knee medial contact force. [36]. This finding supports our hypothesis that subjects classified in the MB phenotype are characterized by disrupted biomechanics. Therefore, interventions aiming to restore an optimal load distribution in the knee such as laterally wedged insoles and gate modification may be more effective if tested on subjects belonging to this phenotype[5].

The I and MD phenotypes showed similar health outcomes. This may be due to the similarity in the hypothesized disease mechanisms. In both these phenotypes, the disease may be due to inflammatory processes. The metabolic disorder characterizing the MD phenotype have been shown to be associated with a low-grade inflammation while synovitis, characterizing the I phenotype, suggests localized more severe inflammation in the knee joint. It may be hypothesized that the involvement of the immune system in the disease process of both these phenotypes may determine similar outcomes. However, these two phenotypes differ in other important characteristics. The I phenotype shows a higher prevalence of synovitis/effusion compared to the MD phenotype which instead presents a higher BMI and a higher prevalence of metabolic alterations. This suggests that despite the similarity in the outcome, the mechanisms of the disease are different and therefore subjects classified in these phenotypes may benefit from different treatments. For example, patients classified in the MS may benefit more from a diet management program and physical activity[37] while subjects with local inflammation may respond better to local steroid injections[38].

The BCM phenotype also had outcomes similar to the I and MD groups. Classification of patients in this phenotype was exclusively done based on high levels of bone and cartilage turnover biomarkers, relative to other patients in the study sample. Among the biomarkers used to classify these subjects, sHA was included. This biomarker is normally associated with synovial activity and may be an indicator of synovitis. However, subjects included in this phenotype showed minimal or no synovitis and effusion at the MRI. High level of sHA in combination with high level of sCOMP (sign of cartilage degradation) may be explained by the release of catabolic enzymes by damaged chondrocytes which degrade the cartilage matrix releasing cartilage degradation products that favour synovial activity[39]. Further study will, therefore, be required to accurately identify the role of specific joint metabolism pathways in the KOA disease process and their viability and validity as separate phenotypes of the disease. Drugs aiming to influence bone and cartilage metabolism may see improved their effect if tested in this specific phenotype[5].

The complex KOA subgroup included all the subjects classified in more than one phenotype. A confluence of disease mechanisms may be responsible for disease outcomes in this group of patients. It was notable that this group reported worse pain and physical functioning than the other phenotype groups. These subjects may require a more complex and multidisciplinary intervention able to target the different mechanism responsible for the disease.

The presence of a group of subjects not classified at the end of the process might imply the existence of further phenotypes whose disease mechanism has not yet been identified or the excessive specificity of the applied cut-offs. Subjects classified in this group present a mild disease with moderate to severe joint damage. This group is primarily similar to the MJD and MB phenotypes. More accurate classification criteria may enable the classification of these patients into those groups.

Despite the fact that the study design cannot directly link these phenotypes with different disease mechanisms; it shows that there are groups of subjects presenting single groups of characteristics previously associated with different disease mechanisms. The CP phenotype was solely defined by symptoms rather than by tissue damage and degeneration. However, in these patients, the severe pain is often associated with a mild involvement of the joint tissue. When defining KOA as a combination of pain and local tissue damage it is clear how, in these patients, the alteration in the pain mechanisms can be considered a major disease mechanism. Therefore, addressing these subjects with tailored interventions aimed to target the specific risk factors and disease mechanisms may increase the effectiveness of the treatments[5].

Some strengths and limitations of this study need to be highlighted. The cut-off values for the sensitive and specific inclusion criteria for phenotype membership used in this study were developed based on evidence extracted from previous studies that focused on the identification of phenotypes and risk factors in the KOA population[6]. Nevertheless, the arbitrary selection of cut-offs could lead to classification bias, as the criteria for inclusion into various phenotypes will not have identical sensitivity and specificity. A two-step classification process was used to ensure that patients clearly showing signs of one particular phenotype were classified in the first (specific) step, meaning that more sensitive criteria could be used to classify the remaining patients without having high rates of false positives for patients clearly belonging to other phenotypes as they had already been classified by that point). This approach is exploratory by nature; further research is needed in order to develop a comprehensive phenotype model applicable in clinical practice. Moreover, any inferences on the prevalence of individual phenotypes can only be made with caution. The combination of cut-off values and the inclusion criteria for this particular OAI cohort are likely to influence the prevalence estimates and their external validity which will have to be replicated in other cohorts.

For the present study, we used the FNIH cohort which was recruited for a case-control study with specific inclusion criteria. The population in exam is characterized in average by a mild to moderate disease where subjects with more severe OA (KL = 4) are underrepresented. Even though the identification of clinical phenotypes in subjects with mild OA may be an advantage for the development of early targeted interventions, it is possible that this classification process would perform differently in a real-life OA population. Further studies performing similar classification processes in clinical cohorts are needed in order to confirm our findings and establish their external validity.

This study utilised a combination of commonly available clinical variables and more advanced measurements. This classification process presents intrinsic advantages and limitations. The use of clinical variables to classify certain phenotypes strengthens the practical relevance of the analyses, while it implies that some of the proposed underlying disease mechanisms were not measured directly. For example, the MB phenotype criteria did not include a direct measurement of knee joint load. The opposite can be said for phenotypes defined using more advanced procedures (e.g. MRI, bone and cartilage biomarkers) which are not routinely performed in the OA patients’ management. This is likely to limit the clinical applicability of this classification process. Further studies are needed to justify the use of such measurements. It is possible that the benefits, both economic and clinical, of phenotype-specific interventions will overcome the costs of these practices increasing their clinical relevance and applicability.

In conclusion, this study has succeeded in classifying groups of subjects characterized by specific clinical characteristics that may be linked to different disease mechanisms and therefore likely to represent distinct clinical phenotypes. However, longitudinal studies analyzing the onset and development of KOA are needed in order to understand whether these clinical characteristics may be considered risk factors for incidence of KOA or the result of different disease paths. In addition, a complex KOA group of patients meeting the inclusion criteria for more than one phenotype was identified. This is a vital step towards the optimization of treatment allocation. While personalized medicine offers great potential to improve treatment effectiveness, the greater need for individual profiling needs to be balanced against the resources available in increasingly stretched healthcare systems worldwide. Identifying homogenous subgroups from within a diagnostic population using clinical information might provide this balance and improve treatment allocation for the individual patients concerned.

## Supporting information

S1 Table Description of the variables used for the classification process. (DOCX)

## Abbreviations

BCM: Bone and Cartilage Metabolism phenotype
BMI: body mass index
CES-D: Center for Epidemiologic Studies Depression scale
CP: Chronic Pain phenotype
I: Inflammatory phenotype
K&L: Kellgren & Lawrence
KOA: Knee Osteoarthritis
MB: Malaligned Biomechanical phenotype
MJD: minimal Joint Disease phenotype
MOAKS: Magnetic Resonance Imaging Osteoarthritis Knee Score
MD: Metabolic Disorder phenotype
sComp: Serum Cartilage Oligomeric Matrix Protein
sHA: Serum Hyaluronic Acid
uCTX-1ß: C-Terminal Telopeptide type 1ß
uCTX-1α: C-Terminal Telopeptide type 1α
uCTXII: C-Terminal Telopeptide type II
uNTXI: Urine N-Terminal Telopeptide type I

## References

[1] Murphy SL, Lyden AK, Phillips K, Clauw DJ, Williams DA. Subgroups of older adults with osteoarthritis based upon differing comorbid symptom presentations and potential underlying pain mechanisms. Arthritis Res Ther. Department of Physical Medicine and Rehabilitation, University of Michigan, 300 North Ingalls, 9th floor, Ann Arbor, MI 48109–2007, USA. sumurphy@umich.edu; 2011;13: R135 doi: 10.1186/ar3449 21864381 10.1186/ar3449 PMC3239378

[2] Andriacchi TP, Favre J, Erhart-Hledik JC, Chu CR. A systems view of risk factors for knee osteoarthritis reveals insights into the pathogenesis of the disease. Ann Biomed Eng. NIH Public Access; 2015;43: 376–87. doi: 10.1007/s10439-014-1117-2 25224078 10.1007/s10439-014-1117-2 PMC4340713

[3] Karsdal M a, Bihlet a., Byrjalsen I, Alexandersen P, Ladel C, Michaels M, et al OA phenotypes, rather than disease stage, drive structural progression–identification of structural progressors from 2 phase III randomized clinical studies with symptomatic knee OA. Osteoarthr Cartil. Elsevier Ltd; 2015;23: 550–558. doi: 10.1016/j.joca.2014.12.024 25576879 10.1016/j.joca.2014.12.024

[4] Driban JB, Sitler MR, Barbe MF, Balasubramanian E. Is osteoarthritis a heterogeneous disease that can be stratified into subsets? Clin Rheumatol. Department of Kinesiology, Temple University, 002 Pearson Hall, 1800 N. Broad Street, Philadelphia, PA 19122, USA. jdriban@temple.edu; 2010;29: 123–131. doi: 10.1007/s10067-009-1301-1 19924499 10.1007/s10067-009-1301-1

[5] Felson DT. Identifying different osteoarthritis phenotypes through epidemiology. Osteoarthritis Cartilage. England; 2010;18: 601–604. doi: 10.1016/j.joca.2010.01.007 20175975 10.1016/j.joca.2010.01.007 PMC3474706

[6] Dell’Isola A., Allan R, Smith SL, Marreiros SSP, Steultjens M. Identification of clinical phenotypes in knee osteoarthritis: a systematic review of the literature. BMC Musculoskelet Disord. BMC Musculoskeletal Disorders; 2016;17: 425 doi: 10.1186/s12891-016-1286-2 27733199 10.1186/s12891-016-1286-2 PMC5062907

[7] Riddle DL, Stratford PW, Perera RA, Emrani PS, Katz JN, Kessler CL, et al The incident tibiofemoral osteoarthritis with rapid progression phenotype: development and validation of a prognostic prediction rule. Osteoarthr Cartil. Elsevier; 2016;24: 2100–2107. doi: 10.1016/j.joca.2016.06.021 27390031 10.1016/j.joca.2016.06.021 PMC5107340

[8] Karsdal MA, Christiansen C, Ladel C, Henriksen K, Kraus VB, Bay-Jensen AC. Osteoarthritis e a case for personalized health care? Osteoarthr Cartil. 2014;22: 7–16. doi: 10.1016/j.joca.2013.10.018 24216058 10.1016/j.joca.2013.10.018

[9] Karsdal MA, Michaelis M, Ladel C, Siebuhr AS, Bihlet AR, Andersen JR, et al Disease-modifying treatments for osteoarthritis (DMOADs) of the knee and hip: lessons learned from failures and opportunities for the future. Osteoarthritis and Cartilage. 2016. pp. 2013–2021. doi: 10.1016/j.joca.2016.07.017 27492463 10.1016/j.joca.2016.07.017

[10] Eckstein F, Wirth W, Nevitt MC. Recent advances in osteoarthritis imaging—the Osteoarthritis Initiative. Nat Rev Rheumatol. 2012;8 doi: 10.1038/nrrheum.2012.113 22782003 10.1038/nrrheum.2012.113 PMC6459017

[11] Hunter DJ, Guermazi A, Lo GH, Grainger AJ, Conaghan PG, Boudreau RM, et al Evolution of semi-quantitative whole joint assessment of knee OA: MOAKS (MRI Osteoarthritis Knee Score). Osteoarthritis Cartilage. 2011;19: 990–1002. doi: 10.1016/j.joca.2011.05.004 21645627 10.1016/j.joca.2011.05.004 PMC4058435

[12] Dekker J, van Dijk GM, Veenhof C. Risk factors for functional decline in osteoarthritis of the hip or knee. Curr Opin Rheumatol. 2009;21: 520–4. doi: 10.1097/BOR.0b013e32832e6eaa 19550331 10.1097/BOR.0b013e32832e6eaa

[13] King CD, Sibille KT, Goodin BR, Cruz-Almeida Y, Glover TL, Bartley E, et al Experimental pain sensitivity differs as a function of clinical pain severity in symptomatic knee osteoarthritis. Osteoarthr Cartil. University of Florida Pain Research and Intervention Center of Excellence (PRICE), FL 32610, USA. kingpininflorida@gmail.com: Elsevier; 2013;21: 1243–1252. doi: 10.1016/j.joca.2013.05.015 23973137 10.1016/j.joca.2013.05.015 PMC3831366

[14] Lewinsohn PM, Seeley JR, Roberts RE, Allen NB. Center for Epidemiologic Studies Depression Scale (CES-D) as a screening instrument for depression among community-residing older adults. Psychol Aging. 1997;12: 277–87. Available: http://www.ncbi.nlm.nih.gov/pubmed/9189988 9189988 10.1037//0882-7974.12.2.277

[15] Waarsing JH, Bierma-Zeinstra SM a., Weinans H. Distinct subtypes of knee osteoarthritis: data from the Osteoarthritis Initiative. Rheumatology (Oxford). Department of Orthopaedics, Department of General Practice, Erasmus Medical Center, Rotterdam, Department of Orthopaedics & Rheumatology, UMC Utrecht and Department of Biomedical Engineering, Delft University of Technology, Delft, The Netherlands e.waarsi:. Published by Oxford University Press on behalf of the British Society for Rheumatology; 2015; 1–9. kev100 [pii] 10.1093/rheumatology/kev100 PMC4536859 25882850

[16] Bae WC, Payanal MM, Chen AC, Hsieh-Bonassera ND, Ballard BL, Lotz MK, et al Topographic Patterns of Cartilage Lesions in Knee Osteoarthritis Cartilage. Department of Radiology, University of California-San Diego, La Jolla, CA; 2010;1: 10–19. doi: 10.1177/1947603509354991 20664706 10.1177/1947603509354991 PMC2909594

[17] Sharma L, Song J, Dunlop D, Felson D, Lewis CE, Segal N, et al Varus and valgus alignment and incident and progressive knee osteoarthritis. Ann Rheum Dis. 2010;69: 1940–1945. doi: 10.1136/ard.2010.129742 20511608 10.1136/ard.2010.129742 PMC2994600

[18] van Spil WEE, Jansen NWDW, Bijlsma JWJW, Reijman M, DeGroot J, Welsing PMJM, et al Clusters within a wide spectrum of biochemical markers for osteoarthritis: data from CHECK, a large cohort of individuals with very early symptomatic osteoarthritis. Osteoarthritis Cartilage. Department of Rheumatology & Clinical Immunology, University Medical Center Utrecht, P.O. Box 85500, 3508 GA, Utrecht, The Netherlands. w.e.vanspil@umcutrecht.nl: Osteoarthritis Research Society International. Published by Elsevier Ltd; 2012;20: 745–754. doi: 10.1016/j.joca.2012.04.004 22503811

[19] Sowers M, Jannausch M, Stein E, Jamadar D, Hochberg M, Lachance L. C-reactive protein as a biomarker of emergent osteoarthritis Osteoarthritis Cartilage. Department of Epidemiology, School of Public Health, University of Michigan, Ann Arbor, Ml 48109–2029, USA mfsowers@umich.edu; 2002;10: 595–601. S1063458402908009 [pii] 12479380 10.1053/joca.2002.0800

[20] van der Esch M, Knoop J, van der Leeden M, Roorda LDD, Lems WFF, Knol DLL, et al Clinical phenotypes in patients with knee osteoarthritis: a study in the Amsterdam osteoarthritis cohort Osteoarthr Cartil. Elsevier Ltd; 2015;23: 544–549. doi: 10.1016/j.joca.2015.01.006 25596322 10.1016/j.joca.2015.01.006

[21] Knoop J, Van Der Leeden M, Thorstensson C a., Roorda LD, Lems WF, Knol DL, et al Identification of phenotypes with different clinical outcomes in knee osteoarthritis: Data from the osteoarthritis initiative. Arthritis Care Res. 2011;63: 1535–1542. doi: 10.1002/acr.20571 21954070 10.1002/acr.20571

[22] Courties A, Gualillo O, Berenbaum F, Sellam J. Metabolic stress-induced joint inflammation and osteoarthritis. Osteoarthritis Cartilage. 2015;23: 1955–65. doi: 10.1016/j.joca.2015.05.016 26033164 10.1016/j.joca.2015.05.016

[23] Courties A, Gualillo O, Berenbaum F, Sellam J, Sellam J, Berenbaum F, et al Metabolic stress-induced joint inflammation and osteoarthritis. Osteoarthr Cartil. Elsevier; 2015;23: 1955–1965. doi: 10.1016/j.joca.2015.05.016 26033164 10.1016/j.joca.2015.05.016

[24] Yoshimura N, Muraki S, Oka H, Tanaka S, Kawaguchi H, Nakamura K, et al Accumulation of metabolic risk factors such as overweight, hypertension, dyslipidaemia, and impaired glucose tolerance raises the risk of occurrence and progression of knee osteoarthritis: a 3-year follow-up of the ROAD study Osteoarthr Cartil. Blackwell Scientific, Oxford; 2012;20: 1217–26. doi: 10.1016/j.joca.2012.06.006 22796312 10.1016/j.joca.2012.06.006

[25] Monira Hussain S, Wang Y, Cicuttini FM, Simpson JA, Giles GG, Graves S, et al Incidence of total knee and hip replacement for osteoarthritis in relation to the metabolic syndrome and its components: a prospective cohort study. Semin Arthritis Rheum. Kinetics Books, Champaign (IL); 2014;43: 429–36. doi: 10.1016/j.semarthrit.2013.07.013 24012045 10.1016/j.semarthrit.2013.07.013

[26] Dahaghin S, Bierma-Zeinstra SMA, Koes BW, Hazes JMW, Pols P. Do metabolic factors add to the effect of overweight on hand osteoarthritis? The Rotterdam Study. Ann Rheum Dis. 2007;66: 916–920. doi: 10.1136/ard.2005.045724 17314121 10.1136/ard.2005.045724 PMC1955104

[27] BERRY PA, MACIEWICZ RA, CICUTTINI FM, JONES MD, HELLAWELL CJ, WLUKA AE. Markers of bone formation and resorption identify subgroups of patients with clinical knee osteoarthritis who have reduced rates of cartilage loss. J Rheumatol. Department of Epidemiology and Preventive Medicine, Monash University, Central and Eastern Clinical School, Alfred Hospital, Melbourne, Victoria 3004, Australia; 2010;37: 1252–1259. doi: 10.3899/jrheum.091055 20395641 10.3899/jrheum.091055

[28] Otterness IG, Swindell a. C, Zimmerer RO, Poole a. R, Ionescu M, Weiner E. An analysis of 14 molecular markers for monitoring osteoarthritis: Segregation of the markers into clusters and distinguishing osteoarthritis at baseline. Osteoarthr Cartil. Inflammation Biology, Pfizer Central Research, Groton, CT 06340, USA. otterx@earthlink.net; 2000;8: 180–185. doi: 10.1053/joca.1999.0288 10806045 10.1053/joca.1999.0288

[29] Roemer FW, Guermazi A, Niu J, Zhang Y, Mohr A, Felson DT. Prevalence of magnetic resonance imaging-defined atrophic and hypertrophic phenotypes of knee osteoarthritis in a population-based cohort. Arthritis Rheum. Department of Radiology, Boston University Medical Center, Boston, Massachusetts 02118, USA. froemer@bu.edu: by the American College of Rheumatology; 2012;64: 429–437. doi: 10.1002/art.33344 22094921 10.1002/art.33344 PMC3268883

[30] Bellamy N, Buchanan WW, Goldsmith CH, Campbell J, Stitt LW. Validation study of WOMAC: a health status instrument for measuring clinically important patient relevant outcomes to antirheumatic drug therapy in patients with osteoarthritis of the hip or knee. J Rheumatol. 1988;15: 1833–40. Available: http://www.ncbi.nlm.nih.gov/pubmed/3068365 3068365

[31] Angst F, Aeschlimann A, Stucki G. Smallest detectable and minimal clinically important differences of rehabilitation intervention with their implications for required sample sizes using WOMAC and SF-36 quality of life measurement instruments in patients with osteoarthritis of the lower ex. Arthritis Rheum. 2001;45: 384–391. doi: 10.1002/1529-0131(200108)45:4<384::AID-ART352>3.0.CO;2-0 11501727 10.1002/1529-0131(200108)45:4<384::AID-ART352>3.0.CO;2-0

[32] Holla JFM, van der Leeden M, Heymans MW, Roorda LD, Bierma-Zeinstra SM a, Boers M, et al Three trajectories of activity limitations in early symptomatic knee osteoarthritis: a 5-year follow-up study. Ann Rheum Dis. Amsterdam Rehabilitation Research Centre, Reade, Amsterdam, The Netherlands.; Amsterdam Rehabilitation Research Centre, Reade, Amsterdam, The Netherlands Department of Rehabilitation Medicine, VU University Medical Centre, Amsterdam, The Netherlands EMGO; 2014;73: 1369–1375. doi: 10.1136/annrheumdis-2012-202984 23716068 10.1136/annrheumdis-2012-202984

[33] Carlesso LC, Segal N, Curtis JR, Wise BL, Law LF, Nevitt M, et al Knee Pain Severity Rather Than Structural Damage is a Risk Factor for Incident Widespread Pain: The Multicenter Osteoarthritis (MOST) Study. Arthritis Care Res (Hoboken). 2016; doi: 10.1002/acr.23086 27636245 10.1002/acr.23086 PMC5354981

[34] Neogi T, Frey-Law L, Scholz J, Niu J, Arendt-Nielsen L, Woolf C, et al Sensitivity and sensitisation in relation to pain severity in knee osteoarthritis: trait or state? Ann Rheum Dis. 2015;74: 682–8. doi: 10.1136/annrheumdis-2013-204191 24351516 10.1136/annrheumdis-2013-204191 PMC4062615

[35] Cruz-Almeida Y, King CD, Goodin BR, Sibille KT, Glover TL, Riley JL, et al Psychological profiles and pain characteristics of older adults with knee osteoarthritis. Arthritis Care Res. 2013;65: 1786–1794. doi: 10.1002/acr.22070 23861288 10.1002/acr.22070 PMC3922880

[36] Dell’Isola A, Smith SL, Andersen MS, Steultjens M. Knee internal contact force in a varus malaligned phenotype in knee osteoarthritis. Osteoarthr Cartil. Osteoarthritis Research Society International; 2017; doi: 10.1016/j.joca.2017.08.010 28882753 10.1016/j.joca.2017.08.010

[37] Messier SP, Mihalko SL, Legault C, Miller GD, Nicklas BJ, DeVita P, et al Effects of Intensive Diet and Exercise on Knee Joint Loads, Inflammation, and Clinical Outcomes Among Overweight and Obese Adults With Knee Osteoarthritis. JAMA. American Medical Association; 2013;310: 1263 doi: 10.1001/jama.2013.277669 24065013 10.1001/jama.2013.277669 PMC4450354

[38] McCabe PS, Parkes MJ, Maricar N, Hutchinson CE, Freemont A, O’Neill TW, et al Synovial Fluid White Cell Count in Knee Osteoarthritis: Association with Structural Findings and Treatment Response. Arthritis Rheumatol (Hoboken, NJ). 2016; doi: 10.1002/art.39829 27482862 10.1002/art.39829 PMC5340187

[39] Heinegård D, Saxne T. The role of the cartilage matrix in osteoarthritis. Nat Rev Rheumatol. 2011;7: 50–6. doi: 10.1038/nrrheum.2010.198 21119607 10.1038/nrrheum.2010.198

