# Supplementary material for: Classification of patients with knee osteoarthritis in clinical phenotypes: Data from the osteoarthritis initiative
Source: PLoS One. 2018 Jan 12;13(1):e0191045. doi: 10.1371/journal.pone.0191045 (PMC5766143; doi:10.1371/journal.pone.0191045)
Supplement: S1 Table — (DOCX) [file pone.0191045.s001.docx]

**Supporting information 1A**

Description of the variables used for the classification process:

| Variable | Variable description |
| --- | --- |
| Self reported worst pain in the last 7 days | Subjects answered to the following question: Please rate the pain that you've had in your right/left knee during the past 7 days by pointing to the number on this card that best describes the pain at its worst. "0" means "No pain" and "10" means "Pain as bad as you can imagine. |
| Kellgren/Lawrence grade | Assessment from standard flexed fixed location (SynaFlexer positioning plexiglass frame) X-rays. Two expert readers independently assessed each X-ray, blinded to each other’s reading and to a subject’s clinical data. Baseline and follow-up X-rays were scored while being viewed simultaneously and with the readers partially blinded to chronological order of the images |
| Depression | Assessed using Centre for Epidemiologic Studies Depression scale (CES-D) score |
| Widespread pain | Subjects answered to the following question: “During the past 30 days, which of these joints have had pain, aching, or stiffness on most days? By most days, we mean more than half the days of a month”.  The patients indicated the painful joint/s using a body chart. |
| Alignment | Alignment was assessed by an experienced musculoskeletal examiner using a hand goniometer. The participant standing facing the examiner. Feet were approximately shoulder-width apart and pointed straight ahead, and weight equally distributed on both feet (Figure 19). Knees and mid-thigh were exposed. The participant fully extended both legs. The centre (hole) of the goniometer was placed on the centre of the knee joint line (found by moving slightly up from the tibial tubercle to the joint line). The lower extendable arm of the goniometer was aligned along the centre of the lower leg below (in line with the patellar tendon) and extended to the centre of the ankle. The upper extendable arm of the goniometer was aligned with mid-thigh above. |
| Cartilage morphology | Cartilage morphology has been assessed using the Magnetic Resonance Imaging Osteoarthritis Knee Score (MOAKS). MRI acquisition was performed using a 3 Tesla MRI system (Trio, Siemens Healthcare, Erlangen, Germany) at the four OAI clinical sites. The MRI pulse sequence protocol included a coronal two-dimensional intermediate-weighted (IW) turbo spin-echo, sagittal three-dimensional (3D) dual-echo at steady-state (DESS), coronal and axial multiplanar reformations of the 3D DESS and sagittal IW fat saturated (FS) TSE sequences. For the MRI acquisition protocol, see the “MRI Manual” link in the operation manuals section of OAI Online at <http://www.oai.ucsf.edu/datarelease/OperationsManuals.asp>.  Each tibiofemoral compartment was divided in three sub-regions: anterior, central and posterior. For the purpose of this study only the central reagion was used. MOAKS scores the size of any cartilage lesions on a 4 point scale based on the percentage of the subregions that the lesion(s) affect. There is also a separate score for the percentage of the subregion that is affected by full thickness cartilage loss. These two scores weere combined into a single number where the portion before the decimal point represents the score for the size of the lesion and the portion after the decimal point represents the score for the amount of full thickness cartilage loss. |
| Diabetes | Diabetes was self-reported using the Charles Comorbidity index. Subjects answerd to the question: “Do you have diabetes (high blood sugar)? |
| Blood and Serum biomarkers | Morning blood and second morning void urine specimens were collected after an overnight fast using a uniform protocol at all clinic visits. The majority of blood was processed and saved as serum for use in biomarker assays for cartilage and bone turnover and proteomic studies and smaller amounts processed for plasma-based assays. Urine specimens were obtained by providing participants with a collection cup and instructions for collecting a second morning void at home on the day of their clinic visit and bringing the specimen to the clinic. Additional details on specimen collection and processing methods can be found in the OAI operations manuals (<http://www.oai.ucsf.edu/datarelease/OperationsManuals.asp>) |
